# Liquid-Chromatographic Methods for Carboxylic Acids in Biological Samples

**DOI:** 10.3390/molecules25214883

**Published:** 2020-10-22

**Authors:** Takuya Fujiwara, Ryoto Inoue, Takuma Ohtawa, Makoto Tsunoda

**Affiliations:** Graduate School of Pharmaceutial Sciences, The University of Tokyo, Tokyo 1130033, Japan; taku1729@gmail.com (T.F.); ryoino888@gmail.com (R.I.); tawashi994865@gmail.com (T.O.)

**Keywords:** fluorescence, mass spectrometry, fatty acids, perfluorinated carboxylic acids, α-keto acids

## Abstract

Carboxyl-bearing low-molecular-weight compounds such as keto acids, fatty acids, and other organic acids are involved in a myriad of metabolic pathways owing to their high polarity and solubility in biological fluids. Various disease areas such as cancer, myeloid leukemia, heart disease, liver disease, and lifestyle diseases (obesity and diabetes) were found to be related to certain metabolic pathways and changes in the concentrations of the compounds involved in those pathways. Therefore, the quantification of such compounds provides useful information pertaining to diagnosis, pathological conditions, and disease mechanisms, spurring the development of numerous analytical methods for this purpose. This review article addresses analytical methods for the quantification of carboxylic acids, which were classified into fatty acids, tricarboxylic acid cycle and glycolysis-related compounds, amino acid metabolites, perfluorinated carboxylic acids, α-keto acids and their metabolites, thiazole-containing carboxylic acids, and miscellaneous, in biological samples from 2000 to date. Methods involving liquid chromatography coupled with ultraviolet, fluorescence, mass spectrometry, and electrochemical detection were summarized.

## 1. Introduction

Quantification of low-molecular-weight compounds, as exemplified by metabolomics studies, has become increasingly important in the life sciences. Metabolite analysis provides metabolic and biochemical status of particular biological systems and valuable insights into disease development and diagnosis [1,2,3,4,5,6]. There are numerous classes of low-molecular-weight compounds, and they are categorized based on their functional groups, including amine, thiol, and carboxylic groups. Low-molecular-weight carboxylic acids are involved in various metabolic pathways. For example, the tricarboxylic acid (TCA) cycle, which is the principal energy-producing process in cells, involves nine carboxylic acid compounds. Fatty acids are integral components of lipids, and consist of carboxylic acids with long aliphatic chains.

Hence, highly sensitive and selective methods for the determination of biologically important carboxylic acids are required for biological investigations, and, thus far, numerous analytical methods have been developed. For selective determination, solid-phase extraction or solvent extraction pretreatment is commonly performed, followed by separation techniques such as liquid chromatography (LC), gas chromatography (GC), and capillary electrophoresis. The choice of detection method is important for trace amounts of carboxylic acids in biological samples. Ultraviolet absorbance detection is rarely implemented due to the absence of chromophores in carboxylic acids. Fluorescence detection following derivatization and mass spectrometry has the advantage of high sensitivity.

This review focuses on analytical methods for carboxylic acids developed since 2000 until March 2020. The aim of this review is to summarize pretreatment and LC separation methods for carboxylic acids in biological samples, such as blood, plasma, urine, and tissue and thus provide a reference for further studies. The review is arranged according to compound classes, namely, fatty acids, TCA cycle and glycolysis-related compounds, amino acid metabolites, perfluorinated carboxylic acids (PFCAs), α-keto acids and their metabolites, thiazole-containing carboxylic acids, and miscellaneous. Detailed analytical conditions for each carboxylic acid class are summarized in Table 1, Table 2, Table 3, Table 4, Table 5, Table 6 and Table 7.

## 2. Analytical Methods for Carboxylic Acids in Biological Samples

### 2.1. Fatty Acids

#### 2.1.1. Analysis of Fatty Acids

**Figure 1:**
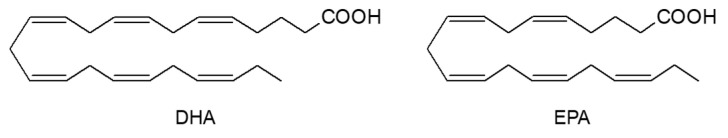
Chemical structures of docosahexaenoic acid (DHA) and eicosapentanoic acid (EPA).

Fatty acids consisting of hydrophobic carbon chains and hydrophilic carboxylic acids are classified into three types depending on the saturation level of the carbon chain moiety (saturated, monounsaturated, and polyunsaturated). A number of fatty acids play critical roles in the body. For example, docosahexaenoic acid (DHA) and eicosapentanoic acid (EPA) (Figure 1), both of which are omega-3 polyunsaturated fatty acids, are not only effective for lowering blood pressure, but are essential for maintaining brain function [85]. Furthermore, fatty acids are related to certain human diseases, such as arteriosclerosis and ischemic heart disease [86,87]. Hence, numerous analytical methods have been developed for fatty acids to elucidate pathological conditions, disease mechanisms, and aid in diagnosis.

Because some fatty acids are unstable at higher temperatures, LC is generally implemented for analysis instead of GC. In addition, physical properties, such as polarity and molecular weight, vary among fatty acids depending on chain length, necessitating optimization according to target compound properties. LC methods for fatty acids in biological samples are classified based on the detection methods described below.

#### 2.1.2. Fluorescence Detection

Fluorescence combined with HPLC is a commonly utilized technique for fatty acids detection, having the advantage of high sensitivity. However, the majority of fatty acids are not fluorescent, necessitating derivatization.

**Figure 2:**
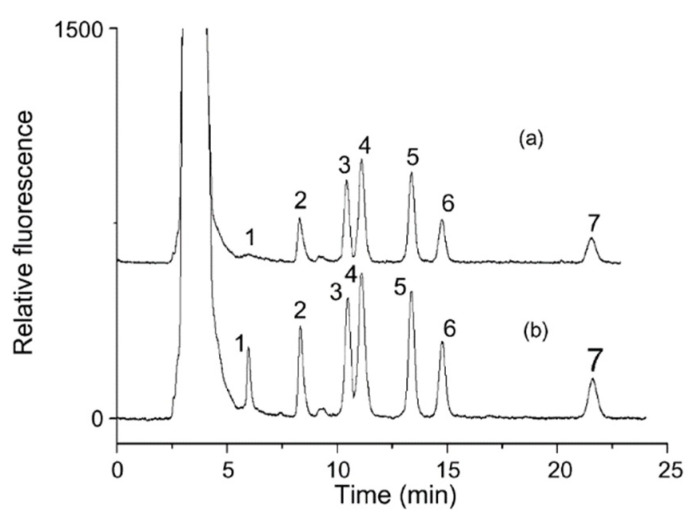
Chromatograms of seven 6-oxy-(acetylpiperazine)fluorescein (APF)-derivatized fatty acids in (**a**) healthy human serum and (**b**) sample containing 0.2 µM fatty acid standards. Peaks: 1, lauric acid; 2, myristic acid, 3, arachidonic acid; 4, linoleic acid; 5, palmitic acid; 6, oleic acid; 7, stearic acid. [7]—Reproduced with the permission of Elsevier.

Du et al. designed 6-oxy-(acetylpiperazine)fluorescein (APF), comprised of a fluorescein fluorophore and a piperazine reactive moiety, for carboxylic acid labeling. As shown in Figure 2, seven fatty acids (lauric, myristic, arachidonic, linoleic, palmitic, oleic, and stearic acid) were determined in human serum [7]. APF has the advantages of a relatively straightforward derivatization procedure, high stability, and sensitivity, wherein LODs of 0.1–6.4 nM are attainable.

2-(2-Naphoxy)ethyl-2-(piperidino)ethanesulfonate (NOEPES) was developed for the quantification of docosaoic (C22), tetracosanic (C24), and hexacosanic (C26) acids [8]. NOEPES is readily removable following derivatization to water-soluble ammonium species, which enables minimal interference when separated by HPLC. The method has been applied to human plasma, and the average levels of C22, C24, and C26 acids were determined to be 566, 398, and 93 nM, respectively.

Other methods have been developed employing 9-(2-hydroxyethyl)-carbazole (HEC) as the derivatization reagent [9,10]. After addition of 1-ethyl-3-(3-dimethylaminopropyl)-carbodiimide (EDC) and 4-dimethylaminopyridine (DMAP) as condensation reagents, derivatization was performed at 60 °C for 30 min. The use of *N,N*’-carbonyldiimidazole (CDI) instead of EDC improved LODs to 38–57 fmol for C14–C20 fatty acids and even lower for <C14 fatty acids.

4-*N,N*-Dimethylaminosulfonyl-7-*N*-(2-aminoethyl)amino-2,1,3-benzoxadiazole (DBD-ED) derivatization was reported by Nishikiori et al. [11]. The LODs were in the range of 2.29–4.75 fmol for free fatty acids, and it was applied to measure fatty acid contents in human serum with acid extraction. Recently, DBD-ED was used for the analysis of EPA and DHA in human serum [12]. The DBD-ED approach is attractive in terms of sensitivity, but it requires a relatively long reaction time (120 min) compared to other derivatization reagents.

Nithipatikom et al. developed a microbore column LC method using 2-(2,3-naphthalimino)ethyl trifluoromethanesulfonate (NT), for the quantification of endogenous epoxyeicosatrienoic acids from endothelial cells at quantities as low as 2 pg [13].

#### 2.1.3. Mass Spectrometry

Mass spectrometry is another common detection method for fatty acids. For ESI-MS detection of fatty acids, the negative mode is often practiced with negatively charged fatty acids. However, in general, the negative mode is less sensitive than the positive.

Bollinger et al. developed a method for fatty acid detection by ESI-MS in the positive mode, using *N*-(4-aminomethylphenyl)pyridinium (AMPP) as a derivatization reagent and accomplished a 64,000-fold increase in sensitivity compared with that of nonderivatized fatty acids [14,15]. Aminoxy tandem mass tags (aminoxyTMTs) have likewise been used for positive mode detection [16]. This method was applied for the quantification of palmitic acid (C16:0) and docosapentaenoic acid (C22:5) in breast cancer cells. In both minimally and highly invasive cancer cells, levels of C22:5 are markedly higher compared to those in benign breast cells. Several benzofurazan derivatization reagents have been developed for positive ESI-MS detection of fatty acids in rat plasma [17,18]. LODs were in the range of 6.5–150 fmol.

**Figure 3:**
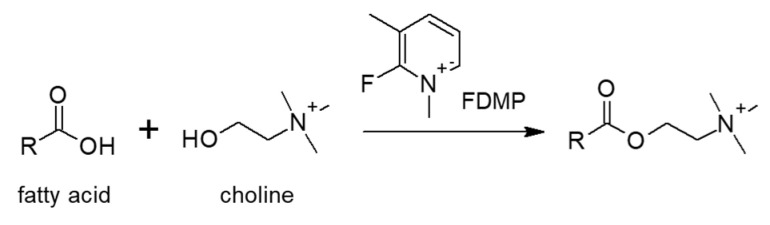
Reaction scheme of fatty acids labeling with choline facilitated by 2-fluoro-1,3-dimethyl-pyridinium (FDMP).

2-Fluoro-1,3-dimethyl-pyridinium (FDMP) is utilized for converting fatty acids to choline derivatives, which remains permanently ionized in both acidic and basic mobile phases, as shown in Figure 3. The LOD for palmitic acid was 50 ng/mL in hydrophilic interaction chromatography (HILIC)-ESI-MS [19].

**Figure 4:**
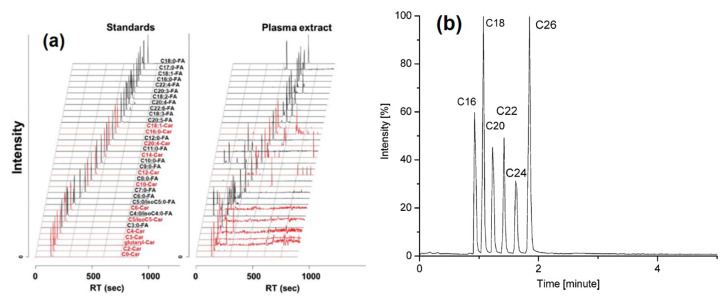
(**a**) LC-MS/MS chromatogram of derivatized fatty acids and acylcarnitines in the standards sample and plasma extract. [20]—Reproduced with the permission of Springer Nature; (**b**) LC-MS chromatogram of six non-derivatized fatty acids. [23]—Copyright (2004) American Chemical Society.

Chen et al. employed dansylhydrazine to label free fatty acids and acylcarnitines resulting in MS detection of 25 fatty acids and 13 acylcarnitines in human plasma within 12 min (Figure 4a) [20]. The derivatization method based on deuterated 2,4-dimethoxy-6-piperazin-1-yl pyrimidine (DMPP) has the advantage of rapid labeling, being executed within 15 sec [21]. 2-Dimethylaminoethylamine (DMED) and d_4_-DMED derivatization enabled the successfully monitoring of metabolic changes in the unsaturated fatty acid biosynthesis pathway in human serum [22]. Nagy et al. employed a partially miscible solvent and stepwise gradient to achieved rapid analyses of fatty acids without derivatization [23]. Figure 4b displays the chromatogram of C16–C26 fatty acids separated via this method within 2 min.

#### 2.1.4. Electrochemical Detection

**Figure 5:**
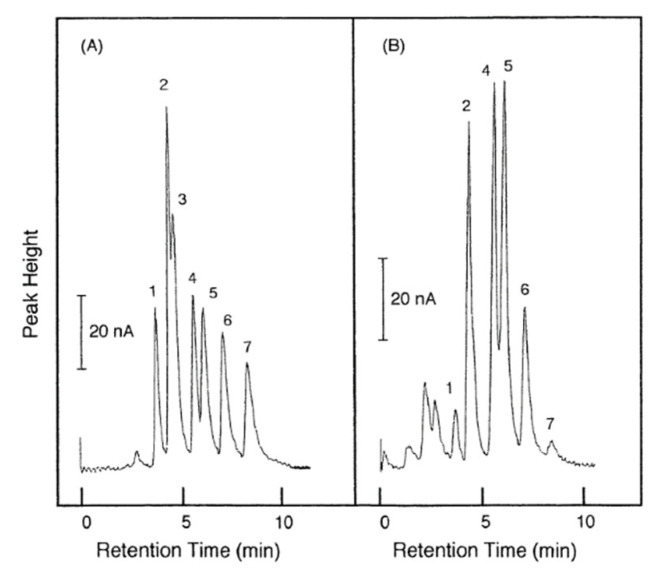
Fatty acid chromatograms of (**A**) sample with standards and (**B**) human serum. Peaks: 1, arachidonic acid; 2, palmitoleic and linoleic acids; 3, myristic acid; 4, oleic acid; 5, palmitic acid; 6, margaric acid (IS); 7, stearic acid. [25]—Reproduced with the permission of Elsevier.

Although fluorescence detection has merit in terms of sensitivity, it suffers several disadvantages, including the requirement for sample pretreatment and derivatization. To overcome these drawbacks, HPLC-electrochemical detection methods have been developed [24,25]. Such protocols require merely 20 min for the complete analysis process of fatty acids without derivatization, wherein separation is achieved within 10 min (Figure 5). The LOD of 50 pmol renders this technique for application in biological samples, such as serum, plasma, and urine.

#### 2.1.5. Electrogenerated Chemiluminescence

An electrogenerated chemiluminescence (ECL) system has been utilized for the sensitive and selective detection of tertiary amine or diketone moieties. Morita et al. extended the HPLC-ECL technique to myristic acid analysis in human plasma with an LOD of 70 fmol by using *N*-(3-aminopropyl) pyrrolidine (NAPP), which converts fatty acids into tertiary amine derivatives [26].

### 2.2. TCA Cycle and Glycolysis-Related Compounds

Glycolysis and the subsequent TCA cycle are the principal metabolic pathways via which ATP is synthesized by substrate level phosphorylation. Metabolic disorders occurring in these pathways are intrinsically and directly associated with numerous diseases, such as diabetes, kidney disease, and cancer [88,89]. The quantification of metabolites is beneficial for clinical diagnoses and quality assurance of organic products. Therefore, simpler, more sensitive and accurate quantification methods have been developed to meet these requirements.

As typified by citric acid, the TCA cycle and glycolysis involve numerous low-molecular-weight high-polarity carboxylic acid metabolites. Moreover, variations in the physical properties of these compounds complicate simultaneous analysis. Therefore, various separation conditions (reversed-phase (RP)LC, ion–pair chromatography, ion–exclusion chromatography, and HILIC), and detection methods (UV, fluorescence, and MS) have been combined to overcome this drawback.

Fumaric acid was quantified with an LC-photodiode array (PDA) to investigate the dynamics of fumaric acid-constitutive drug nanocarriers [27]. Trace levels of maleic acid in healthy rat serum and urine were analyzed with LC-MS/MS [28]. Methylmalonic acid in human plasma was quantified by HILC-MS [29], lactic acid in human urine and saliva with LC-UV and fluorescence detection after fluorescence derivatization (two detection methods were compared) [30], and oxalic acid in mouse urine and hepatocyte samples with ion–exclusion chromatography–MS/MS [31]. These methods were developed for sensitive and selective detection with simple operation.

**Figure 6:**
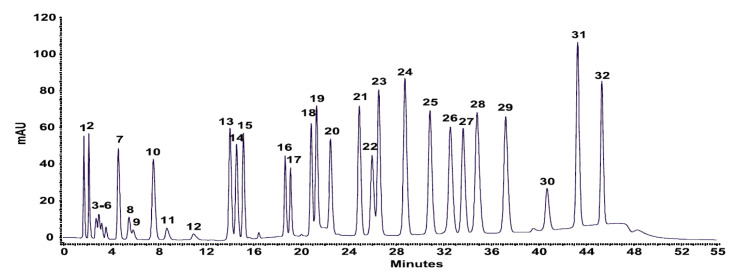
Chromatogram of simultaneous separation of carboxylic acids under single-run chromatographic conditions. Peaks: 1, oxalic; 2, tartaric; 3, malic; 4, malonic; 5, lactic; 6, acetic; 7, maleic; 8, citric; 9, succinic; 10, fumaric; 11, propionic; 12, levulinic; 13, methylsuccinic; 14, pyromellitic; 15, gallic; 16, protocatechuic; 17, 3,5-dihydroxybenzoic; 18, trimellitic; 19, phthalic; 20, 4-hydroxybenzoic; 21, 2,4-dihydroxybenzoic; 22, vanillic; 23, syringic; 24, 2-methoxybenzoic; 25, trimesic; 26, benzoic; 27, ferulic; 28, salicylic; 29, 3-methoxybenzoic; 30, 2-methylbenzoic; 31, cinnamic; 32, 3-methoxycinnamic acid. [34]—Reproduced with the permission of Springer Nature.

Simultaneous analysis of numerous carboxylic acid metabolites has been performed by several groups. Kubota et al. quantified six compounds via 4-*N,N*-dimethylaminosulfonyl-7-piperazino-2,1,3-benzoxadiazole (DBD-PZ) derivatization and fluorescence detection [32]. Ion exclusion chromatography–UV detection was implemented for the quantification of 9 and 32 carboxylic acid metabolites, to obtain carboxylic acid profiles of cultured yeast samples [33] and human urine [34]. Figure 6 illustrates the separation of 32 carboxylic acid standards with single-run chromatographic conditions.

Similarly, comprehensive analyses including that of carboxylic acid metabolites of the TCA cycle and glycolysis have been conducted with various separation and detection methods: 13 compounds via HPLC-fluorescence detection aided by 1-pyrenemethylamine derivatization [35], 30 compounds with HILIC or ion-pair chromatography-MS/MS [36], 59 compounds with ion-pair chromatography-MS [37], and 138 compounds with UHPLC-MS/MS [38]. Nemkov et al. comprehensively analyzed TCA cycle metabolites to elucidate the effects of hypoxia on carboxylic acid metabolism in red blood cells [39].

### 2.3. Amino Acid Metabolites

Amino acid analysis is immensely important, as they are essential structural units of protein. Recent analytical methods for the quantification of amino acids have been summarized in our previous review [90]. These methods contribute to the diagnosis and elucidation of disease mechanisms, such as diabetes, kidney disease, and liver disease. Measurement of amino acid metabolite levels is likewise beneficial for understanding diseases.

**Figure 7:**
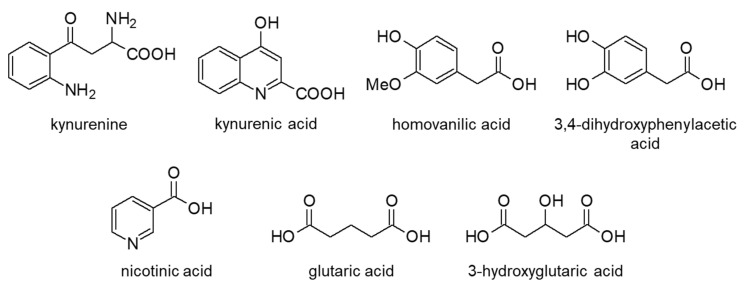
Chemical structures of amino acid metabolites.

Among amino acid metabolites, tryptophan and tyrosine metabolites are most commonly analyzed (Figure 7). Kynurenine and kynurenic acid, which are tryptophan metabolites that are intricately related to diseases such as schizophrenia [91], Parkinson’s disease, and Alzheimer’s disease [92], have been quantified using HPLC-fluorescence detection. Indole derivatives and glycated tryptophan in rat plasma, mouse plasma, and brain were analyzed with fluorescence detection [40,41], and those in livestock urine and plasma were analyzed with LC-MS to monitor their health [42,43,44]. Valko-Rokytovska et al. quantified melanin-related carboxylic compounds in human urine by HPLC-UV for the diagnosis of melanoma cancer, and found that homovanilic acid and tryptophan levels increased in the initial clinical stage of the disease [45]. Catechol-bearing carboxylic acids were separated by ion exchange chromatography, followed by derivatization with ethylenediamine and fluorescence detection [46]. The concentrations of dopamine metabolites, 3,4-dihydroxyphenylacetic and homovanillic acid were determined as 131 and 404 nM in rat kidney samples, and the LODs per injection were 50 and 100 fmol, respectively. Huang et al. quantified nicotinic acid in human plasma by LC-MS/MS to examine its side effects, as it is administered as a potent vitamin at milligram doses [47].

Glutaric and 3-hydroxyglutaric acid were analyzed in urine samples by UHPLC-MS/MS via derivatization with 4-[2-(*N,N*-dimethylamino)ethylaminosulfonyl]-7-(2-aminoethylamino)-2,1,3-benzoxadiazole (DAABD-AE), capable of selectively reacting with dicarboxylic compounds [48]. Levels of both compounds increased significantly in glutaric acidemia type I patients, and it was concluded that glutaric acid analysis was beneficial for the precise diagnosis of the disease.

**Figure 8:**
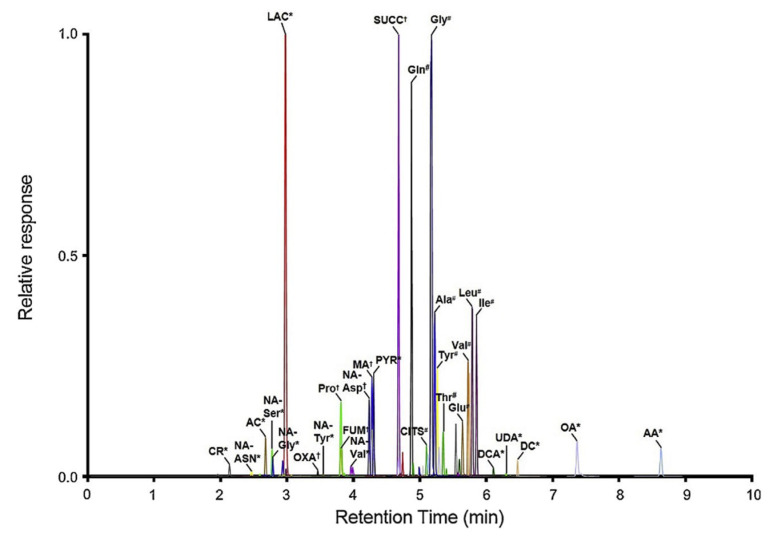
LC–MS/MS chromatogram of 64 metabolites after dimethylaminophenacyl bromide (DmPABr) derivatization in SUIT-2 cells. DmPA labeling patterns are also included (* = labeled once; † = labeled twice; # = labeled thrice). [49]—Reproduced with the permission of Elsevier.

Comprehensive analysis of amino acids, N-acetylated amino acids, and other organic acids in human urine and human pancreatic cancer cells was conducted via derivatization with dimethylaminophenacyl bromide (DmPABr) for MS/MS [49]. DmPABr labels carboxylic acids, thiols, and amines simultaneously, resulting in the appearance of several labelling patterns (Figure 8). The LODs of a total of 64 compounds ranged between 0.11 and 2192 nM, and, importantly, it was below 10 nM for approximately half of them.

### 2.4. Perfluorinated Carboxylic Acids

Perfluorinated carboxylic acids (PFCAs) are synthetic substances used in numerous products such as food packaging paper and impregnation sprays. They are widespread organic pollutants and have been reported to accumulate in the human body because they are resistant to metabolism [93]. Although critical adverse effects of PFCAs on human health have not been reported, evaluation of their accumulation in the body is critical for further investigation of PFCA effects.

Several sample pretreatments, such as solid-phase extraction (SPE), liquid–liquid extraction (LLE), or solvent precipitation can be performed, the most common method being SPE. Maestri et al. analyzed perfluorooctanoic acid using a C18 disposable SPE cartridge [50]. Oasis WAX cartridges, wherein extraction is conducted in a mixed mode of RP and weak anion exchange, have likewise been used [51,52]. Prior to SPE operation, a mixture of HCl and HNO_3_ was added to eliminate the matrix, in the case of bivalve samples, leading to improved recoveries (92–104%) and selectivity. The developed method was applied to shell and soft tissues, and no significant relationship was found between PFCA levels in shell and soft tissues.

**Figure 9:**
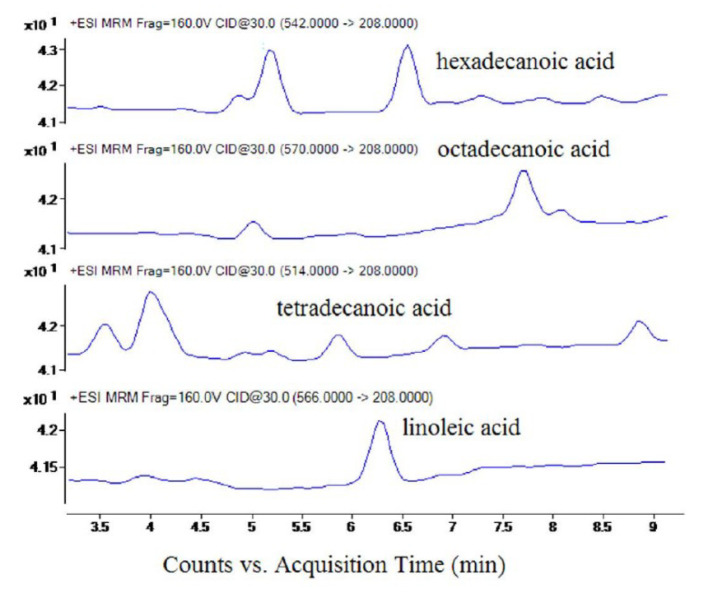
LC-MS/MS chromatograms of 10-methyl-acridone-2-sulfonohydrazide (MASH)-derivatized PFCAs in serum samples. [55]—Reproduced with the permission of Elsevier.

Gao et al. established an online SPE-LC-MS/MS method for 21 per- and polyfluoroalkyl substances including 13 PFCAs, featuring a rapid processing time (20 min per sample) and favorable peak shapes [53]. Perfluorobutanoic and perfluorooctanoic acid concentrations were found to be significantly higher in the serum of employees of fluorochemical manufacturing plants than those in the general population. Micro-solid phase extraction (µ-SPE) can determine trace levels of PFCAs in human plasma [54]. Zhang et al. precipitated proteins using acetonitrile and derivatized PFCAs with 10-methyl-acridone-2-sulfonohydrazide (MASH) to eliminate the matrix effect [55]. Chromatograms of MASH-derivatized PFCAs in serum samples are shown in Figure 9.

### 2.5. α-Keto Acids and Their Metabolites

#### 2.5.1. α-Keto Acids

**Figure 10:**
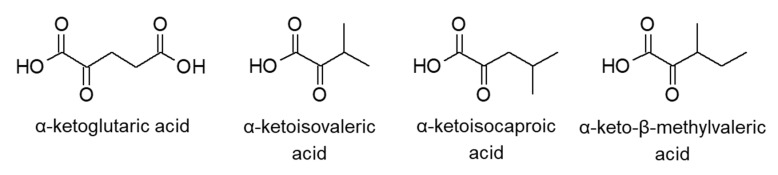
Chemical structures of α-keto acids.

α-Keto acids are synthesized from α-amino acids via transamination. Some biological α-keto acids (α-ketoglutaric acid, α-ketoisovaleric acid, α-ketoisocaproic acid, and α-keto-β-methylvaleric acid, Figure 10) are assembled from α-amino acids by branched-chain amino acid transferase (BCAT), and others are likewise found in amino acid metabolic pathways. We found that BCAT1 plays a significant role in the development of chronic myeloid leukemia [94]. In addition, disorders of α-keto acid levels in biological fluids are related to diseases such as diabetes mellitus, maple syrup urine disease, and ketoacidosis [95,96]. Therefore, the analysis of α-keto acids is attracting increasing interest.

As α-keto acids are not fluorescent, their detection was accomplished with fluorescence detection after derivatization. Derivatization reagents such as *o*-phenylenediamine (OPD) and 1,2-diamino-4,5-methylenedioxybenzene (DMB) can execute an α-keto-acid-selective reaction through the recognition of two conjugated carbonyl groups.

**Figure 11:**
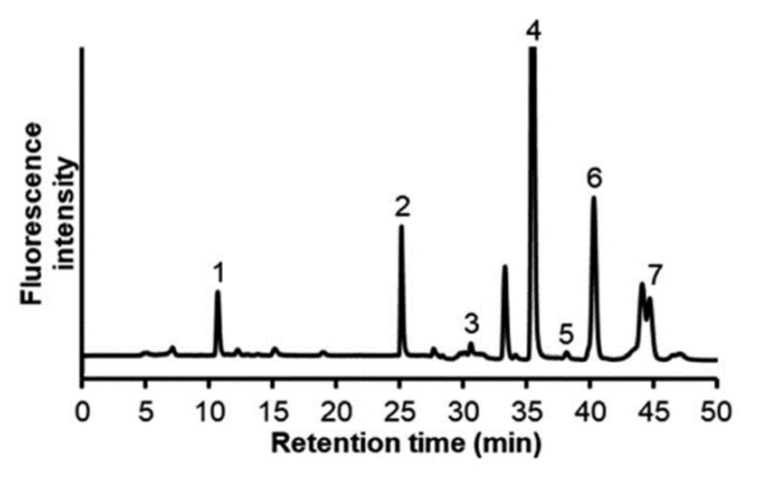
Chromatogram of 1,2-diamino-4,5-methylenedioxybenzene (DMB)-α-keto acids in K562 cell sample. Peaks: 1, DMB-α-ketoglutaric acid; 2, DMB-pyruvic acid; 3, DMB-α-ketobutyric acid, 4, DMB-α-ketvaleric acid; 5, DMB-α-ketoisovaleric acid; 6, DMB-α-ketoisocaproic acid; 7, DMB-α-keto-β-methylvaleric acid. [61]—Reproduced with the permission of The Royal Society of Chemistry.

Quantification entailing OPD derivatization and fluorescence detection has been reported by several groups, wherein LODs were between 18 nM and 1 µM [58,59,60]. DMB was developed to improve the sensitivity of fluorescence detection, and has a structure similar to that of OPD. As shown in Figure 11, we quantified α-keto acids in chronic myeloid leukemia cells to investigate the role of BCAT1 [61]. The concentrations of six α-keto acids were between 1.55 and 316 pmol per 106 cells and LODs were within 1.3 and 5.4 nM, being three-fold higher than that of OPD.

OPD derivatization was also employed for MS, resulting in a lowered LOD of 5 nM. Tissue samples from PP2Cm knockout mice had 22–86 times higher branched-chain α-keto acids than wild-type mice [62]. Ten α-keto acids were analyzed with *O*-(2,3,4,5,6-pentafluorobenzyl)oxime (*O*-PFBO) derivatization and MS/MS. The method was applicable to the comprehensive analysis of α-keto acids in rat plasma [63]. Li et al. analyzed branched-chain α-keto acids and α-amino acids simultaneously by MS/MS without derivatization and found significant differences in the concentrations of six compounds in patients with cerebral infarction and those of healthy individuals [64].

#### 2.5.2. 2-Hydroxyglutaric Acid

**Figure 12:**
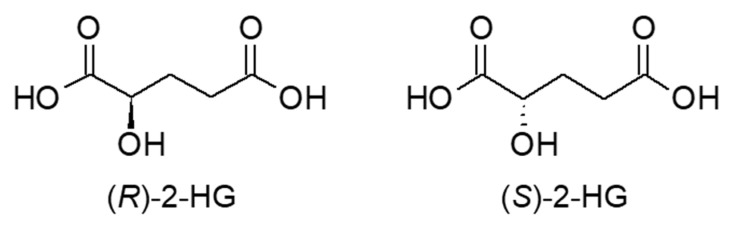
Chemical structures of (**R**)- and (**S**)-2-hydroxyglutaric acid (2-HG).

**Figure 13:**
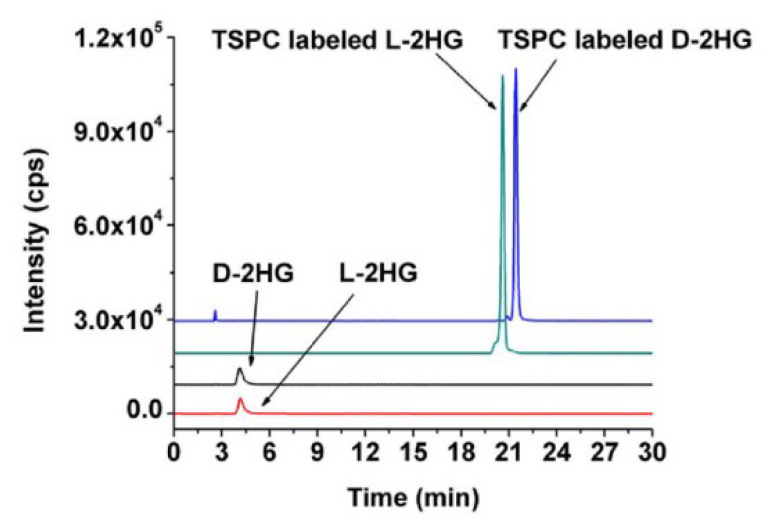
LC-MS/MS chromatograms of non-labeled and *N*-(*p*-toluenesulfonyl)-L-phenylalanyl chloride (TSPC)-labeled (***R***)- and (***S***)-2-HG (**D** and **L**, respectively). [66].

2-Hydroxyglutaric acid (2-HG, Figure 12) is an oncometabolite produced from α-ketoglutaric acid. Because 2-HG is chiral and only (*R*)-2-HG is a cancer metabolite, it is necessary to separate the enantiomeric isomers. One of the strategies for separating enantiomeric isomers is the chiral derivatization method. (+)-*O,O’*-Diacetyl-L-tartaric anhydride (DATAN) [65] and *N*-(*p*-toluenesulfonyl)-L-phenylalanyl chloride (TSPC) [66] are two reagents developed for this purpose. Both reagents have been used for RPLC-MS/MS analysis. Whereas TSPC was superior to DATAN in sensitivity (LOD of (*R*)-2-HG was 1.2 fmol with TSPC and 115 fmol with DATAN), DATAN enabled faster separation than TSPC (23 min for TSPC, 6 min for DATAN). Figure 13 illustrates the separation of enantiomers by TSPC derivatization [66]. It was revealed that levels of both (*R*)- and (*S*)-2-HG in human urine were comparable among patients with type 2 diabetes mellitus, lung cancer, colorectal cancer, and nasopharyngeal carcinoma. On the other hand, both isomers of 2-HG in human tissue were significantly increased.

### 2.6. Carboxylic Acids Containing a Thiazole Ring

**Figure 14:**
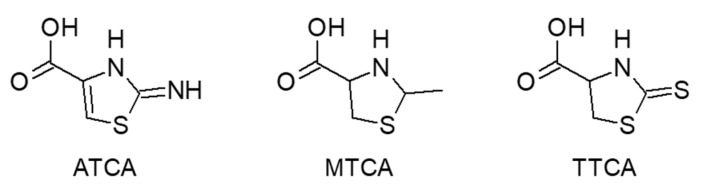
Chemical structures of 2-aminothiazoline-4-carboxylic acid (ATCA), 2-methylthiazolidine-4-carboxylic acid (MTCA), and 2-thiothiazolidine-4-carboxylic acid (TTCA).

2-Aminothiazoline-4-carboxylic acid (ATCA, Figure 14) is a cyanide metabolite and is expected to be a biomarker of cyanide-related diseases due to its thermal and long-term stability in biological samples. In forensic cases, whole blood is the preferred biological sample, and it is necessary to purify ATCA from blood because of its high viscosity and complex composition. Purification methods using cation-exchange SPE [67] and molecularly imprinted polymer-stir bar sorptive extraction (MISBSE) [68] have been proposed, to be followed by LC-MS/MS. SPE offered adequate precision of 5.9% RSD, but interferences derived from whole blood were detected near the ATCA peak. On the other hand, MIP-SBSE produced a single peak derived from ATCA, but the capacity was insufficient and the method suffered from low recoveries [69]. Lulinski et al. synthesized a novel imprinted material in a dispersive SPE, providing a rapid (35 min for extraction) and low-cost clean-up technique [70]. After investigation of the surface morphology, interferences were significantly reduced. A robust SPE was developed for hydrophilic interactions, allowing for a high accuracy of 96% [71]. Furthermore, it is environmentally benign and entails a simple procedure. Analysis of human postmortem blood from individuals who died due to oral cyanide exposure established that ATCA is an adequate cyanide exposure marker.

Although LLE offered good accuracy and sensitivity with an LOD of 0.43 ng/mL, derivatization of interference compounds (secondary amino acids) was required to remove interference peaks [72]. It was applied to human blood and revealed that ATCA concentrations in blood from a cyanide-poisoned person were approximately four-fold higher than those from non-poisoned persons.

2-Methylthiazolidine-4-carboxylic acid (MTCA, Figure 14) is produced by the reaction of acetaldehyde and cysteine, and it is a biomarker for recent alcohol consumption. MTCA has been derivatized with acetic anhydride and then analyzed by reversed-phase LC-ESI-MS [73]. Free MTCA is unstable under physiological conditions as it is readily hydrolyzed. Hence, the quantification of MTCA was enabled by N-acetylation.

2-Thiothiazolidine-4-carboxylic acid (TTCA, Figure 14) is a urinary metabolite derived from carbon disulfide. Methyl t-butyl ether and diethyl ether have been utilized as extraction solvents for TTCA, the former being superior in terms of TTCA recovery (78–87% against 67–80%) as well as storage stability as it produces less peroxides during storage [74]. After extraction, TTCA was analyzed by reversed-phase LC-UV.

### 2.7. Miscellaneous

Other biological carboxylic acids not discussed in the above sections are described here. The detection method was optimized for the physical properties of each compound.

**Figure 15:**
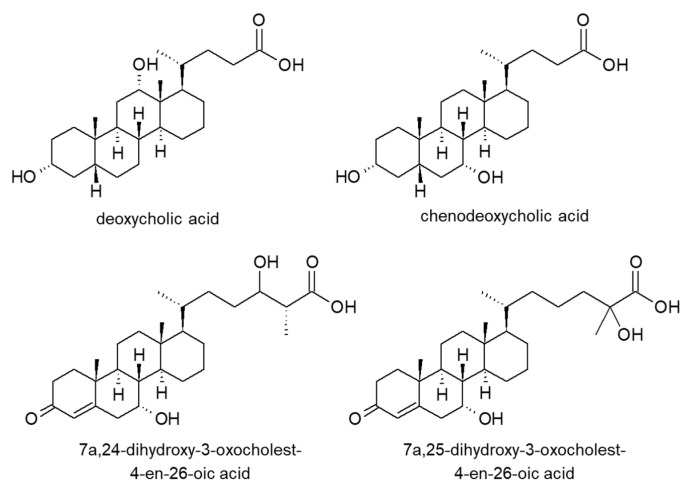
Chemical structures of bile acids and their metabolites.

Bile acids and their metabolites have been analyzed using several different derivatization reagents. Higashi et al. developed an analytical method employing 2-picolylamine derivatization and MS/MS detection and monitored these compounds in human saliva [75]. Sensitivity in MS/MS was improved by approximately ten-fold by 1-(3-aminopropyl)-3-bromoquinolinium bromide (APBQ) derivatization [76]. Concentrations of deoxycholic acid and chenodeoxycholic acid (Figure 15) in human plasma were 91 and 445 nM, respectively. 2-(7H-Dibenzo[a,g]carbazol-7-yl)ethyl 4-methylbenzenesulfonate (DBCETS) derivatization was also performed for fluorescence detection to determine bile acid concentrations in human serum [77]. Derivatization with 2-bromo-4′-nitroacetophenone and phenacyl bromide was also used for UV detection [78,79].

As intermediates of bile acid biosynthesis, dihydroxyoxocholestenoic acids were quantified using LC-MS [80]. It was found that concentrations of 7α,24- and 7α,25-dihydroxy-3-oxocholest-4-en-26-oic acids (Figure 15) in cerebrospinal fluid were reduced in patients with hereditary spastic paraplegia type 5. Tetrahydroglucocorticoid glucuronides in human urine were quantified with 1-[(4-dimethylaminophenyl)-carbonyl]piperazine (DAPPZ) to diagnose diseases caused by abnormal cortisol secretion [81].

Orotic acid, which is useful for diagnosing urea cycle disorder or hereditary orotic aciduria, was analyzed by LC-MS/MS. with an LOD of 150 nM [82].

Metabolome analysis is challenging because a variety of carboxylic compounds with a wide range of concentrations in complex biological samples need to be simultaneously detected. Guo et al. implemented isotope-labeled DmPABr derivatization and LC-MS, resulting in the identification of 51 carboxylic compounds in urinary samples [83]. Isotope-labeled dansylhydrazine was also used to identify 81 compounds in the urinary sample [84]. The research was conducted as a demonstration; therefore, further application of the method is expected.

## 3. Conclusions

In this review, LC methods for the quantification of carboxylic acids in biological samples, such as plasma, serum, and urine, were summarized. In general, a practical analytical method is achieved when several aspects are promoted: high sensitivity, high selectivity, rapid analysis, minimal reagent and equipment requirements, and simple operation. Numerous analytical methods have been developed to improve upon the existing ones.

The most commonly employed detection methods include fluorescence and MS. Fluorescence detection achieves a relatively better detection limit with simple equipment, but required fluorescence derivatization. For MS analysis, various types of compounds could be analyzed simultaneously without derivatization. Derivatization reagents for MS have been used not only for improved separation but also for enhancing sensitivity and selectivity. In addition, short-time analyses have been developed with optimized derivatization reagents and separation conditions.

Practical and efficient analytical methods are continually being developed to meet the requirements of quantifying various compounds in diverse samples. The utilization of these methods is beneficial for elucidating disease mechanisms, and further contributions to disease diagnosis and treatment are anticipated.

## Figures and Tables

**Table 1:** Analytical methods for fatty acids in biological samples.

Target Compounds	Biological Sample	Sample Treatment	Derivatization Reagent	Separation Mode	Detection Method	LOD	Recovery	Ref.
7 Fatty acids	Human serum	Acid extraction	APF	RPLC	FL: 467/512 nm	0.1–6.4 nM	93–105%	[7]
3 Fatty acids	Human plasma	Acid extraction	NOEPES	RPLC	FL: 235/366 nm	56 fmol	–	[8]
6 Fatty acids	Human plasma	Acid extraction	HEC	RPLC	FL: 293/365 nm	38–57 fmol	102–106%	[9]
6 Fatty acids	Human plasma	Acid extraction	HEC	RPLC	FL: 335/365 nm	45–68 fmol	102–105%	[10]
5 Fatty acids	Human serum	Acid extraction	DBD-ED	RPLC	FL: 450/560 nm	2.29–4.75 fmol	108–113%	[11]
8 Fatty acids	Rat plasma	Acid extraction	DBD-ED	RPLC	FL: 450/560 nm	–	–	[12]
4 Epoxyeicosatrienoic acids	Bovine endothelial cells	Solid phase extraction	NT	RPLC	FL: 259/395 nm	<2 pg	83–89%	[13]
25 Fatty acids	Mouse serum	Acid extraction	AMPP	RPLC	MS/MS	50–100 fg (LOQ)	–	[14]
11 Fatty acids	Mouse serum, bronchial epithelial cells	Solid phase extraction	AMPP	RPLC	MS/MS	200–900 fg (LOQ)	–	[15]
20 Fatty acids	Breast cancer cells	Solvent extraction	Aminoxy TMT	RPLC	MS/MS	40 fmol	–	[16]
8 Fatty acids	Rat plasma	Acid extraction	DBD-PZ-NH_2_	RPLC	MS	<0.1 µM	–	[17]
9 Fatty acids	Rat plasma	Solvent extraction	DAABD-AE	RPLC	MS	6.5–21 fmol	–	[18]
			MePZBD-AE	RPLC	MS	8.8–32 fmol	–	[18]
			APZBD-NHMe	RPLC	MS	35–150 fmol	–	[18]
56 Fatty acids	Human plasma	Centrifugation	Choline	HILIC	MS	50 ng/mL	–	[19]
38 Fatty acids, acylcarnitines	Human plasma	Centrifugation	Dansyl-hydrazine	RPLC	MS/MS	76–152 pM	–	[20]
18 Fatty acids	Human urine	Solid phase extraction	d_0_-DMPP, d_6_-DMPP	RPLC	MS/MS	5–15 pM	–	[21]
60 Fatty acids	Human serum	Acid extraction	DMED, d_4_-DMED	RPLC	MS	–	–	[22]
6 Fatty acids	Human blood	Acid extraction	None	RPLC	MS	low pg range	–	[23]
4 Fatty acids	Human serum, plasma	Solvent extraction	None	RPLC	ECD	50 pmol	92–102%	[24]
6 Fatty acids	Human plasma	Solvent extraction	None	RPLC	ECD	50 pmol	92–102%	[25]
11 Fatty acids	Human plasma	Solvent extraction	AEMP, NAPP	RPLC	Electrogenerated chemiluminescence	70 fmol	–	[26]

APF: 6-oxy-(acetyl piperazine)fluorescein, NOEPES: 2-(2-naphoxy)ethyl 2-(piperidino)ethanesulfonate, HEC: 9-(2-hydroxyethyl)-carbazole, DBD-ED: 4-*N,N*-dimethylaminosulfonyl-7-*N*-(2-aminoethyl)amino-2,1,3-benzoxadiazole, NT: 2-(2,3-naphthalimino)ethyl trifluoromethanesulfonate, AMPP: *N*-(4-aminomethylphenyl)pyridinium, AminoxyTMT: aminoxy tandem mass tags, DBD-PZ-NH_2_: 7-(*N,N*-dimethylaminosulfonyl)-4-(aminoethyl)piperazino-2,1,3-benzoxadiazole, DAABD-AE: 4-[2-(*N,N*-dimethylamino)ethylaminosulfonyl]-7-(2-aminoethylamino)-2,1,3-benzoxadiazole, MePZBD-AE: [4-(4-*N*-methyl)piperazinosulfonyl]-7-(2-aminoethylamino)-2,1,3-benzoxadiazole, APZBD-NHMe: [4-(4-*N*-aminoethyl)piperazinosulfonyl]-7-methylamino-2,1,3-benzoxadiazole, DMPP: 2,4-dimethoxy-6-piperazin-1-yl pyrimidine, DMED: 2-dimethylaminoethylamine, AEMP: 2-(2-aminoethyl)-1-methylpyrrolidine, NAPP: *N*-(3-aminopropyl)pyrrolidine.

**Table 2:** Analytical methods for TCA cycle and glycolysis-related compounds in biological samples.

Target Compounds	Biological Sample	Sample Treatment	Derivatization Reagent	Separation Mode	Detection Method	LOD	Recovery	Ref.
Fumaric acid	Rat liver, spleen and urine	Centrifugation	None	RPLC	PDA: 215 nm	0.01 µg	89–92%	[27]
Maleic acid	Rat serum and urine	Centrifugation	None	RPLC	MS/MS	0.2 µg/L	94–111%	[28]
Methylmalonic acid	Human plasma	Centrifugation	None	HILIC	MS	0.03 µM	90–93%	[29]
Lactic acid	Human urine and saliva	Centrifugation	9-CMA	RPLC	UV: 365 nm, FL: 365/410 nm	50 nM	92–106%	[30]
Oxalic acid	Mouse urine and hepatocyte	Centrifugation	None	Ion exclusion chromatography	MS/MS	2 µM	–	[31]
6 TCA metabolites	Rat urine	Centrifugation	DBD-PZ	RPLC	FL: 450/560 nm	2–15 fmol	80–96%	[32]
9 Organic acids	Yeast	Centrifugation	None	Ion exclusion chromatography	UV: 210 nm	0.6–29.3 g/L	98–103%	[33]
32 Organic acids	Human urine	Solvent extraction	None	Ion exclusion chromatography	UV: 220 nm	0.002–2.2 g/L	–	[34]
13 Organic acids	Mouse urine	Centrifugation	1-Pyrene methylamine	RPLC	FL: 345/375, 345/475 nm	4–22 fmol	–	[35]
30 Organic acids	Mouse serum, urine, and tissue	Centrifugation	None	HILIC, Ion pair RPLC	MS/MS	<5 µM	–	[36]
59 Organic acids	Human melanoma cells	Centrifugation	Phenylhdrazine	Ion pair RPLC	MS	–	–	[37]
138 Organic acids	Yeast	Centrifugation	None	RPLC	MS/MS	0.001–3.7 µM	–	[38]
TCA metabolites	Human red blood cell	Centrifugation	None	RPLC	MS	–	–	[39]

9-CMA: 9-chloromethyl anthracene, DBD-PZ: 7-(*N,N*-dimethylaminosulfonyl)-4-piperazino-2,1,3-benzoxadiazole.

**Table 3:** Analytical methods for amino acid metabolites in biological samples.

Target Compounds	Biological Sample	Sample Treatment	Derivatization Reagent	Separation Mode	Detection Method	LOD	Recovery	Ref.
Kinurenic acid	Rat plasma	Centrifugation	None	RPLC	FL: 251/398 nm	0.16 nM	97–98%	[40]
3 Trp metabolites	Mouse plasma and brain	Centrifugation	None	RPLC	UV, FL	0.03–1.33 µM	83–116%	[41]
6 Trp metabolites	Pig urine, plasma	Centrifugation	None	RPLC	MS	10–100 ng/mL (LOQ)	–	[42]
Glycated Trp	Chicken plasma	Solvent extraction	None	RPLC	MS	–	–	[43]
PHP-THβC	Chicken plasma	Cation-exchange resin	None	RPLC	MS	–	–	[44]
5 Trp and Tyr metabolites	Human urine	Centrifugation	None	RPLC	UV: 220, 280 nm, FL: 280/350, 315/425 nm	–	–	[45]
DOPAC, HVA	Rat kidney	Microdialysis	Ethylenediamine	Ion exchange chromatography	FL: 417/495 nm	50, 100 fmol	–	[46]
Nicotinic acid	Human plasma	Solvent extraction	None	RPLC	MS/MS	6.57 ng/mL (LOQ)	70–72%	[47]
Glutaric acid, 3-HG	Human urine	Centrifugation	DAABD-AE	RPLC	MS/MS	20–25 nM	94–121%	[48]
64 amino acid derivatives	Human urine, pancreatic cancer cells	Centrifugation	DmPABr	RPLC	MS/MS	0.11–2192 nM	–	[49]

PHP-THβC: (1*R*, 3*S*)-1-(D-gluco-1, 2, 3, 4, 5-pentahydroxypentyl)-1,2,3,4-tetrahydro-β-carboline-3-carboxylic acid, DOPAC: 3,4-dihydroxyphenylacetic acid, HVA: homovanillic acid, 3-HG: 3-hydroxyglutaric acid, DAABD-AE: 4-[2-(*N,N*-dimethylamino)ethylaminosulfonyl]-7-(2-aminoethylamino)-2,1,3-benzoxadiazole, DmPABr: dimethylaminophenacyl bromide.

**Table 4:** Analytical methods for perfluorinated carboxylic acids (PFCAs) in biological samples.

Target Compounds	Biological Sample	Sample Treatment	Derivatization Reagent	Separation Mode	Detection Method	LOD	Recovery	Ref.
3 PFASs	Human tissues and blood	Solid phase extraction	None	RPLC	MS	3 µg/L	80–101%	[50]
10 PFASs	Two bivalves shells, soft tissues	Solid phase extraction	None	RPLC	MS/MS	0.05–0.43 ng/g	92–104%	[51]
18 PFASs	Human urine and serum	Solid phase extraction	None	RPLC	MS/MS	0.1 µg/L	94–104%	[52]
21 PFASs	Human serum	Solid phase extraction	None	RPLC	MS/MS	0.008–0.19 µg/L	85–114%	[53]
6 PFASs	Human plasma	µ-SPE	None	RPLC	MS/MS	21–65 ng/L	88–102%	[54]
6 PFASs	Human serum	Deproteinization	MASH	RPLC	MS/MS	0.07–0.42 µg/L	96–100%	[55]
11 PFASs	Human blood	Solvent extraction	None	RPLC	MS/MS	0.06–0.14 µg/L	67–112%	[56]
20 PFASs	Human plasma, BCS	Centrifugation	None	RPLC	MS/MS	0.024–0.096 µg/L (LOQ)	83–103%	[57]

PFASs: polyfluoroalkyl substances, MASH: 10-methyl-acridone-2-sulfonohydrazide.

**Table 5:** Analytical methods for α-keto acids and 2-hydroxyglutaric acid (2-HG) in biological samples.

Target Compounds	Biological Sample	Sample Treatment	Derivatization Reagent	Separation Mode	Detection Method	LOD	Recovery	Ref.
4 α-Keto acids	Human serum	Centrifugation	OPD	RPLC	FL: 350/410 nm	1 µM	86–109%	[58]
7 α-Keto acids	Human neutrophil	Centrifugation	OPD	RPLC	FL: 360/415 nm	0.035–0.125 µM	79–108%	[59]
3 α-Keto acids	Human CML cell	Gel extraction	OPD	RPLC	FL: 360/415 nm	18–40 nM	84–96%	[60]
6 α-Keto acids	Human CML cell	Centrifugation	DMB	RPLC	FL: 367/446 nm	1.3–5.4 nM	86–118%	[61]
3 α-Keto acids	Mouse tissue	Acid extraction	OPD	RPLC	MS	5 nM	76–95%	[62]
10 α-Keto acids	Rat plasma	Centrifugation	*O*-PFBO	RPLC	MS/MS	0.01–0.25 µM	96–109%	[63]
3 α-Keto acids	Human plasma	Centrifugation	None	RPLC	MS/MS	0.04 µg/mL	81–98%	[64]
(*R*)-2-HG	Human serum	Solid phase extraction	DATAN	RPLC	MS/MS	0.060 µM	31–32%	[65]
(*R*)-2-HG	Human urine, cancer tissues	Solvent extraction	TSPC	RPLC	MS/MS	1.2 fmol	88–109%	[66]

OPD: *o*-phenylenediamine, DMB: 1,2-diamino-4,5-methylenedioxybenzene, *O*-PFBO: *O*-(2,3,4,5,6-pentafluorobenzyl)oxime, DATAN: (+)-*o,o*’-diacetyl-l-tartaric anhydride, TSPC: *N*-(*p*-toluenesulfonyl)-L-phenylalanyl chloride.

**Table 6:** Analytical methods for 2-aminothiazoline-4-carboxylic acid (ATCA), 2-methylthiazolidine-4-carboxylic acid (MTCA), and 2-thiothiazolidine-4-carboxylic acid (TTCA) in biological samples.

Target Compounds	Biological Sample	Sample Treatment	Derivatization Reagent	Separation Mode	Detection Method	LOD	Recovery	Ref.
ATCA	Rat plasma and organ	Solid phase extraction	None	RPLC	MS/MS	–	–	[67]
ATCA	Human urine	MISBSE	None	RPLC	MS/MS	5 µg/L	–	[68]
ATCA	Rat plasma	Solid phase extraction	None	RPLC	MS/MS	12 µg/L	–	[69]
ATCA	Human postmortem blood	Solid phase extraction	None	HILIC	MS/MS	2.5 µg/L	81–89%	[70]
ATCA	Human postmortem blood	Solid phase extraction	None	HILIC	MS/MS	9 µg/L (LOQ)	88–96%	[71]
ATCA	Human postmortem blood	Liquid-liquid extraction	None	HILIC	MS/MS	0.43 µg/L	86–101%	[72]
MTCA	Human blood and urine	Centrifugation	Acetic anhydride	RPLC	MS/MS	0.1 mg/L	–	[73]
TTCA	Urine	Acid extraction	None	RPLC	UV: 271 nm	35 µg/L	78–87%	[74]

MISBSE: molecularly imprinted stir bar sorption extraction.

**Table 7:** Analytical methods for other carboxylic acids in biological samples.

Target Compounds	Biological Sample	Sample Treatment	Derivatization Reagent	Separation Mode	Detection Method	LOD	Recovery	Ref.
7 Bile acids	Human saliva	SPE and solvent extraction	2-Picolylamine	RPLC	MS/MS	1.5–5.6 fmol	–	[75]
3 Bile acids, 8 fatty acids	Human plasma and saliva	Solid phase extraction	APBQ	RPLC	MS/MS	0.19–0.51 fmol	–	[76]
7 Bile acids, 9 fatty acids	Human serum	Solvent extraction	DBCETS	RPLC	FL: 300/395 nm	0.28–0.70 ng/mL	92–102%	[77]
4 Bile acids	*C. bovis*	Centrifugation	2-bromo-4′-nitroacetophenone	RPLC	UV: 263 nm	0.25–0.31 ng	94–99%	[78]
7 Bile acids	Human feces	Solid phase extraction	Phenacyl bromide	RPLC	UV: 254 nm	1.22–1.46 pmol	72–102%	[79]
	Human feces	Solid phase extraction	None	PRLC	MS/MS	–	–	[79]
Dihydroxyoxocholestenoic acids	Human CSF and plasma	Solid phase extraction	Isotope-labeled Girard’s P Reagent	RPLC	MS	0.02–0.05 ng/mL	–	[80]
7 THGC glucuronides	Human urine	Centrifugation	Isotope-labeled DAPPZ	RPLC	MS/MS	0.008–0.16 µg/mL (LOQ)	–	[81]
Orotic acid	Urine	Dilution	None	RPLC	MS/MS	0.15 µM	–	[82]
Metabolome	Human urine	Centrifugation	Isotope-labeled DmPABr	RPLC	MS	–	–	[83]
Metabolome	Human urine	Centrifugation	Isotope-labeled dansylhydrazine	RPLC	MS	–	–	[84]

APBQ: 1-(3-aminopropyl)-3-bromoquinolinium bromide, DBCETS: 2-(7H-dibenzo[a,g]carbazol-7-yl)ethyl 4-methylbenzenesulfonate, DAPPZ: 1-[(4-dimethylaminophenyl)-carbonyl]piperazine, DmPABr: dimethylaminophenacyl bromide.
